# Transcriptional profiling of human macrophages during infection with *Bordetella pertussis*

**DOI:** 10.1080/15476286.2020.1727694

**Published:** 2020-02-19

**Authors:** Denisa Petráčková, Mariam R. Farman, Fabian Amman, Irena Linhartová, Ana Dienstbier, Dilip Kumar, Jakub Držmíšek, Ivo Hofacker, Maria Eugenia Rodriguez, Branislav Večerek

**Affiliations:** a Institute of Microbiology of the Czech Academy of Sciences, Laboratory of Post-transcriptional Control of Gene Expression, Prague, Czech Republic; b Institute for Theoretical Chemistry, University of Vienna, Vienna, Austria; c Division of Cell and Developmental Biology, Medical University of Vienna, Vienna, Austria; d Institute of Microbiology of the Czech Academy of Sciences, Laboratory of Molecular Biology of Bacterial Pathogens, Prague, Czech Republic; e Faculty of Computer Science, Research Group Bioinformatics and Computational Biology, University of Vienna, Vienna, Austria; f Facultad de Ciencias Exactas, Universidad Nacional de La Plata, CINDEFI (UNLP CONICET La Plata), La Plata, Argentina

**Keywords:** *Bordetella pertussis*, infection, macrophage, intracellular survival, host-pathogen interaction

## Abstract

*Bordetella pertussis*, a strictly human re-emerging pathogen and the causative agent of whooping cough, exploits a broad variety of virulence factors to establish efficient infection. Here, we used RNA sequencing to analyse the changes in gene expression profiles of human THP-1 macrophages resulting from *B. pertussis* infection. In parallel, we attempted to determine the changes in intracellular *B. pertussis*-specific transcriptomic profiles resulting from interaction with macrophages. Our analysis revealed that global gene expression profiles in THP-1 macrophages are extensively rewired 6 h post-infection. Among the highly expressed genes, we identified those encoding cytokines, chemokines, and transcription regulators involved in the induction of the M1 and M2 macrophage polarization programmes. Notably, several host genes involved in the control of apoptosis and inflammation which are known to be hijacked by intracellular bacterial pathogens were overexpressed upon infection. Furthermore, *in silico* analyses identified large temporal changes in expression of specific gene subsets involved in signalling and metabolic pathways. Despite limited numbers of the bacterial reads, we observed reduced expression of majority of virulence factors and upregulation of several transcriptional regulators during infection suggesting that intracellular *B. pertussis* cells switch from virulent to avirulent phase and actively adapt to intracellular environment, respectively.

## Introduction

*Bordetella pertussis* is a Gram-negative strictly human pathogen of the respiratory tract and the aetiological agent of whooping cough [1]. Despite vaccination programmes, pertussis remains one of the 10 most common causes of vaccine-preventable deaths [2]. Furthermore, pertussis incidence is currently on the rise in industrialized countries with highly vaccinated populations [3,4]. The re-emergence of pertussis strongly suggests that we need to widen our understanding of the molecular mechanisms underlying the pathogenicity of *B.pertussis* [5,6].

Upon infection, *B. pertussis* cells are challenged by the host innate immune system. While ciliated epithelial cells secrete mucus that mechanically eliminates the bacteria, resident as well as attracted phagocytic cells interact with the pathogen in order to clear the infection [7]. Successful infection, therefore, depends on activities of a great variety of virulence factors produced by *B. pertussis* cells [8]. In particular, adhesins such as filamentous haemagglutinin and fimbriae and toxins such as pertussis toxin and adenylate cyclase toxin contribute to efficient adhesion and colonization of the epithelium as well as to modulation of the immune system and thereby facilitate the survival of bacteria in the respiratory tract [9]. *B. pertussis* was historically described as an extracellular pathogen, which, in the presence of specific antibodies, can be internalized and killed by phagocytic cells [10]. Nevertheless, some early reports suggested that in the absence of opsonization this pathogen stimulates its own attachment to immune cells resulting in inefficient killing by phagocytes and increased intracellular survival [11–15]. Importantly, *B. pertussis* cells were found also within human, rabbit, and mouse alveolar macrophages *in vivo* [16–18]. Within the last decade, Rodriguez and her team presented data indicating that *B. pertussis* can survive in primary macrophages [19]. They have shown that the majority of *B. pertussis* cells are killed within acidic compartments; however, some residual cells evade this killing and survive and replicate inside a non-acidic early endosome-like compartment [20,21]. Indeed, *B. pertussis* uses several mechanisms to avoid phagocytic killing. For example, immune evasion mediated by both pertussis and adenylate cyclase toxins results in subversion of cellular signalling, repression of phagocytic activity and, eventually, in induction of apoptosis [22–25]. Collectively, these data led to speculations that *B. pertussis* could use macrophages as an intracellular niche and that the intramacrophage phase of infection could play a significant role in survival and persistence of bacteria within the host [12,17,18]. In support, gene expression profiling and proteomic analysis of infected monocyte-derived THP-1 macrophages revealed that intracellular *B. pertussis* cells modulate expression of several genes involved in response to the host immune system and reshape production of dozens of proteins implicated in virulence [20,21].

In this study, representing the first attempt to assess the global response of human phagocytes to *B. pertussis* infection, we aimed at RNA-seq analysis of transcriptomic profiles in monocyte-derived THP-1 macrophages infected with *B. pertussis*. In addition, we were tempted to decipher whether we can also monitor the pathogen gene expression profiles during the infection to gain insight into its response and adaptation to the intracellular environment.

## Results

### *Global transcriptomic profile of THP-1 macrophages infected with* B. pertussis

**Figure 1.**
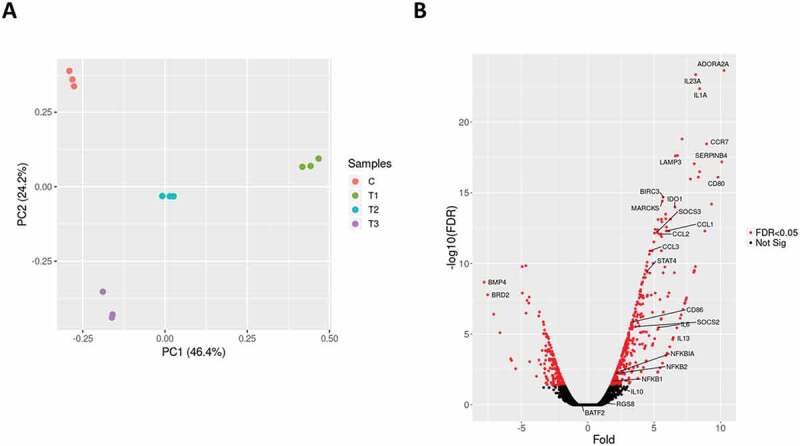
(A) Principal component analysis was applied to transcriptomic profiles of the uninfected THP-1 macrophages (C; red circles) and *B. pertussis*-infected THP-1 macrophages harvested 2 h (T1, green circles), 6 h (T2, blue circles) and 24 h (T3, cyan circles) pi. Each dot represents an independent biological replicate. (B) Volcano plot showing the global transcriptional changes in the infected THP-1 cells 6 h pi. The red dots represent significantly differentially expressed genes, labelled genes are discussed in this work. The black dots represent nonsignificantly modulated genes.

THP-1 monocyte-derived macrophages were infected with *B. pertussis* reference strain Tohama I. Samples of uninfected macrophages (C; control) were collected 6 h post-infection (pi) while infected macrophages were collected at three time points (T1-T3) corresponding to 2, 6 and 24 h pi with the objective of a) elucidation of the general macrophage response triggered by *B. pertussis* (6 h pi) and b) monitoring the time-dependent changes in gene expression profiles resulting from the host–pathogen interaction within the early phase of infection (T2 versus T1) and within the late phase of infection (T3 versus T2). We did not use the uninfected controls for T1 and T3 time points as the changes in gene expression profiles of THP-1 macrophages incubated for 24 h in RPMI medium are not substantial [26] and in particular, expression of several cytokines and chemokines remains unchanged during 24 h incubation in RPMI medium [27,28]. RNA-seq analysis of samples from infected cells and uninfected control yielded on average 16 million reads which were mapped to human and *B. pertussis* genomes (Fig S1). Principal component analysis (PCA) of RNA-seq data revealed that samples from uninfected macrophages clustered separately from infected cells and that also samples of infected macrophages collected at 2 h, 6 h and 24 h pi clustered apart from each other (Fig. 1A). Thus, PCA indicated temporal changes in gene expression profiles resulting from *B. pertussis* infection. To identify these alterations, differential expression (DE) analysis was performed. First, we compared global gene expression profiles in infected cells and control uninfected cells harvested 6 h pi (T2 versus C). For the host, DE analysis identified 679 macrophage genes as significantly modulated (│log_2_FC│ ≥ 1; adjusted p-value < 0.05) upon infection (Table S1). Expression of hundreds of genes was strongly upregulated upon infection including those encoding cytokines, chemokines, cell receptors and transcription factors (Fig. 1B).

Many of the highly expressed genes encode important factors of the innate immunity such as IL-1A, IL-6, IL-23A, TNF superfamily proteins, CCL and CXCL chemokine families and NFκB and STAT4 regulators and belong to common expression pattern mounted by host cells upon bacterial infection [29]. Of note, among the significantly upregulated genes were also anti–inflammatory cytokines *IL-13* and *IL-10* and *ADORA2A* gene encoding the adenosine receptor A_2A_ which senses adenosine levels and triggers the anti–inflammatory signalling [30]. On the other side, among the highly downregulated genes were *BMP4* and *BRD2*. BMP4 belongs to the TGF-β superfamily of proteins and was reported to induce M2 polarization in macrophages [31], while transcriptional regulator BRD2 interacts with chromatin and plays important role in inflammatory response in mice [32]. Collectively, these results suggest that both pro-inflammatory and anti–inflammatory programmes that presumably result from host-pathogen interplay are active in infected cells.

**Figure 2.**
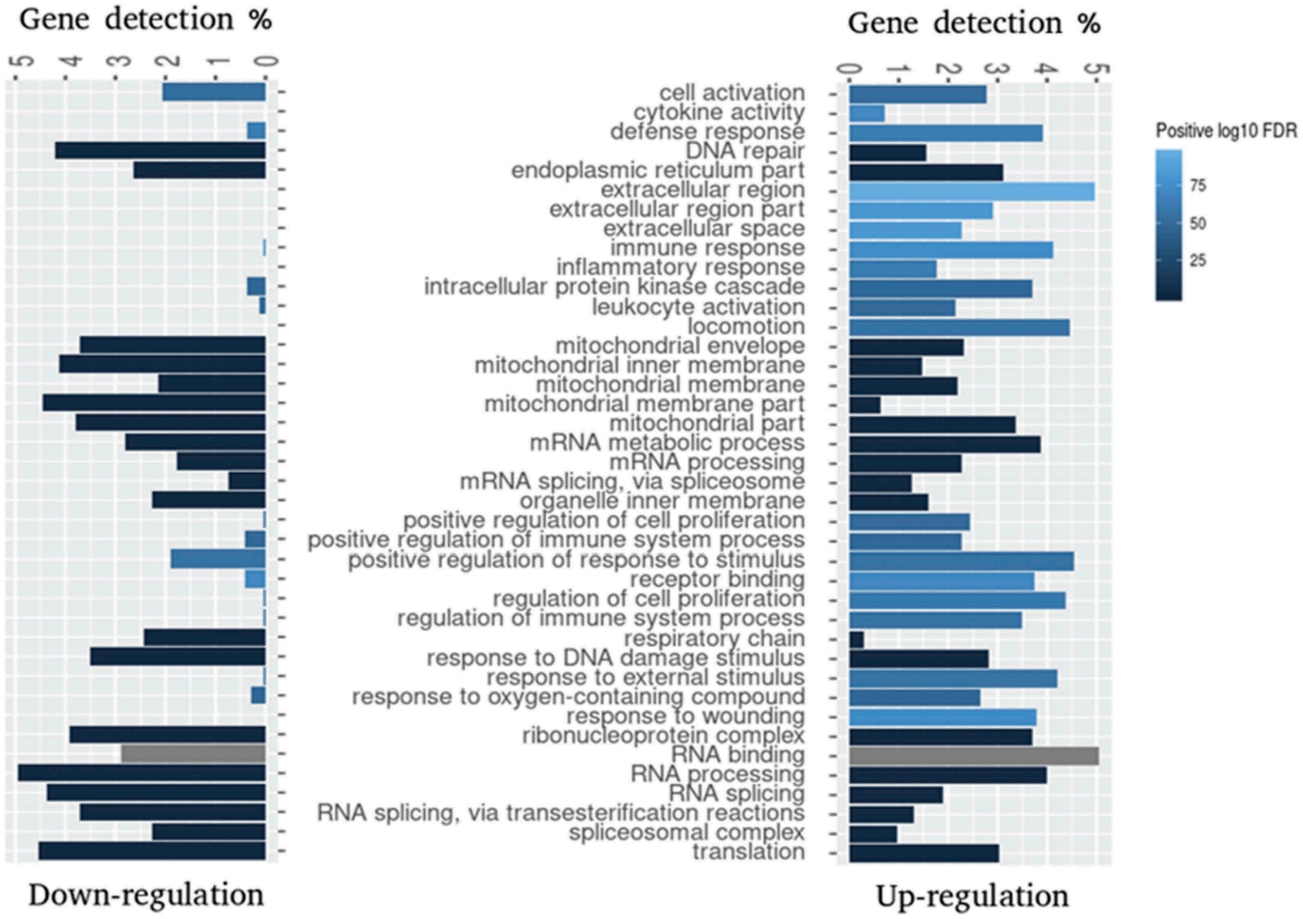
Gene Set Enrichment Analysis (GSEA) of GO terms enriched for differentially expressed genes 6 h pi. GSEA was applied to identify enriched GO terms within either downregulated (log_2_FC < 0) or upregulated (log_2_FC > 0) gene sets. The bars depict the percentage of DE genes associated with the specific GO term and shades of blue indicate positive log_10_ of FDR range. Grey bars indicate that this GO term was not significantly enriched within the corresponding gene set. Top 20 enriched gene sets were selected for visualization.

**Figure 3.**
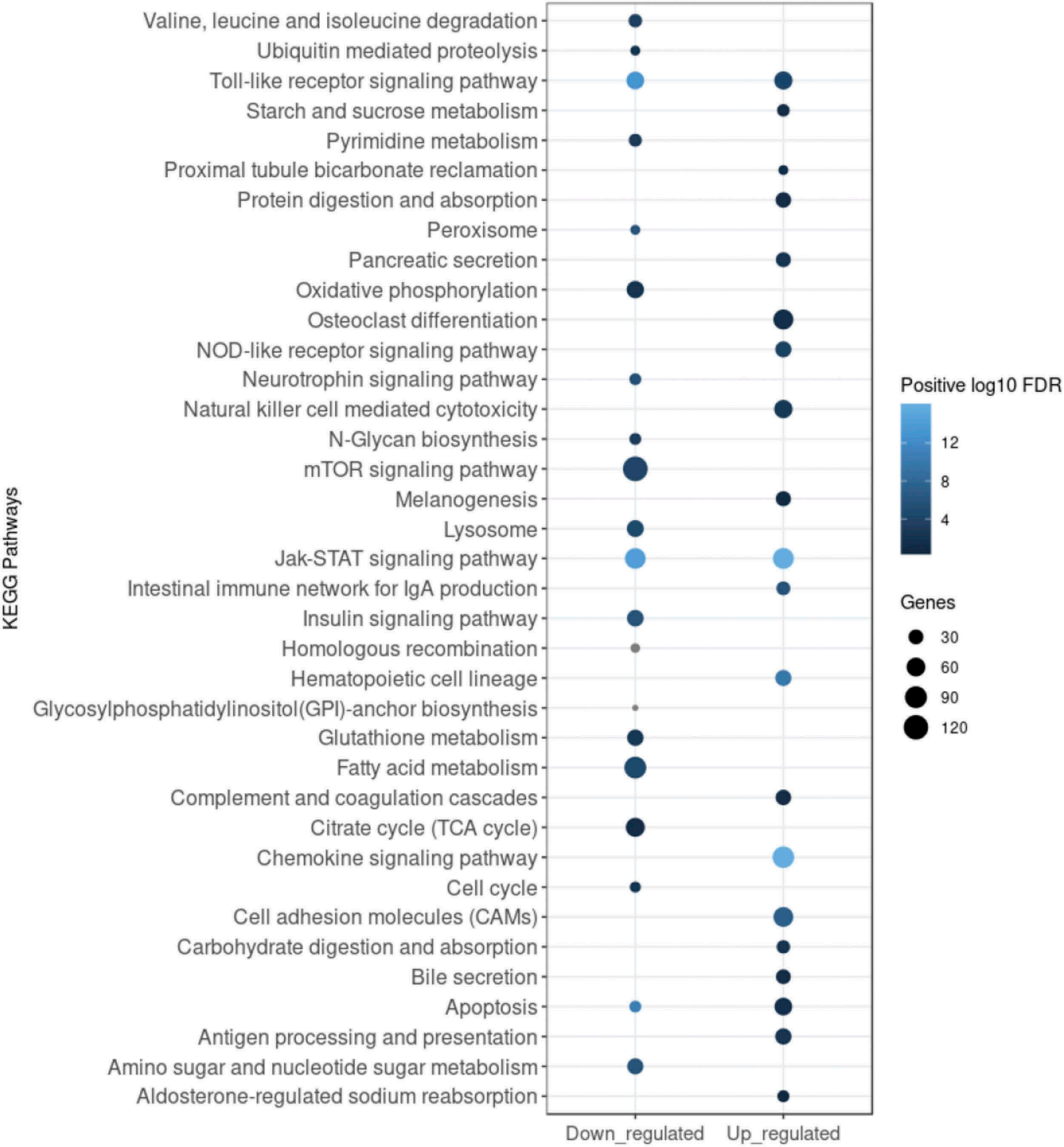
Enrichment analysis of KEGG pathways enriched for DE genes 6 h pi. Analysis was applied to identify pathways enriched for either downregulated (log_2_FC < 0) or upregulated (log_2_FC > 0) DE genes. Top 20 enriched KEGG pathways for each subset of genes were selected for visualization. The legend shows the positive log_10_ of FDR range (shades of blue) and the number of DE genes in each KEGG pathway expressed by the size of the dots.

To obtain a deeper insight into the functional clustering of up- and downregulated DE genes, we performed GO term and KEGG pathway enrichment analyses. As shown in Fig. 2, the Gene Set Enrichment Analysis (GSEA) revealed that genes upregulated upon infection were significantly and selectively enriched within GO terms such as ‘Response to external stimulus’, ‘Immune response’, ‘Leukocyte activation’, ‘Locomotion’ and ‘Regulation of immune system process’, while downregulated genes were enriched predominantly within ‘Respiratory chain’ and ‘Mitochondrial membrane part’ terms. KEGG pathway analysis presented in Fig. 3 identified several regulatory circuits to be selectively enriched for upregulated genes such as ‘NOD-like receptor signalling pathway’, ‘Chemokine signalling pathway’or ‘Antigen processing and presentation’. On the other side, ‘mTOR signalling pathway’ and several metabolic pathways were enriched for downregulated genes.

**Figure 4.**
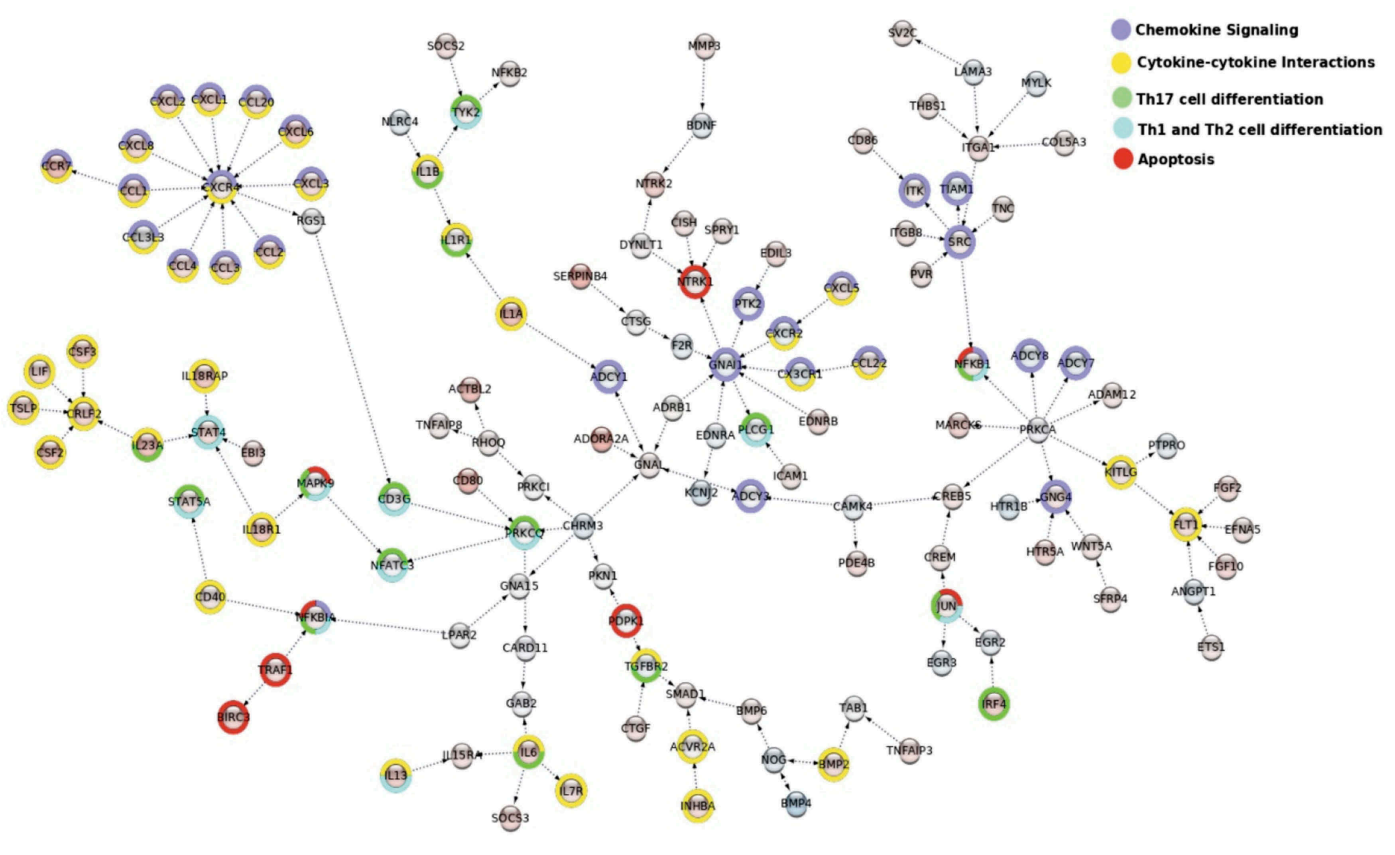
MSF identification of modulated interacting networks. Networks were generated using data from DE analysis. The network file created by MSF was used to visualize the modulated networks in Cytoscape. The nodes represent the genes and the edges show the direction of interaction. The enrichment within different KEGG pathways (see legend) is shown by the colours around the nodes. The colouring inside the node depicts the range of log fold change for upregulated (shades of red) and downregulated genes (shades of blue).

Finally, to identify coherently modulated genes within the global cell signalling network we used the Modulated Sub-graph Finder (MSF) tool. MSF combines the whole cell signalling network with data from DE analysis to identify the modulated networks. The networks identified by MSF help to reveal the flow and cross-talk between different KEGG pathways. As presented in Fig. 4, the selected network shows the signalling between cytokine and chemokines affecting Th1, Th2 and Th17 profiling and apoptosis in immune cells. For example, the graph depicts activation of STAT4 by IL-23 and IL-18R1 or activation of CXCR4 receptor by members of CCL and CXCL chemokine families.

### Temporal changes in global transcriptomic profiles of infected THP-1 macrophages

Next, we focused on time-dependent changes in transcriptomic profiles of infected macrophages. We compared the gene expression profiles in cells harvested 6 h and 2 h pi (T2 versus T1) and in cells harvested 24 h and 6 h pi (T3 versus T2). DE analysis identified 561 and 553 of significantly (│log_2_FC│ ≥ 1; adjusted p-value < 0.05) modulated genes between T2-T1 and T3-T2 time points, respectively (Table S2). Significantly up- and downregulated genes were used for GSEA to identify enriched GO terms between T2-T1 and T3-T2 time points (Fig. S2). Apparently, most of the GO terms were not selectively enriched for either up- or downregulated genes. Nevertheless, as shown in Fig. S3a, when we compared DE genes modulated between T2 and T1 time points, KEGG pathway analysis unravelled several pathways consistently enriched for upregulated (e.g. ‘Phagosome’, ‘Cell adhesion molecules’ and ‘Antigen processing and presentation’) or downregulated (e.g. ‘DNA replication’, ‘Ubiquitin mediated proteolysis’ and ‘Insulin signalling pathway’) genes. Of note, several pathways such as ‘MAPK signalling pathway’, ‘Toll-like receptor pathway’ and ‘Hedgehog signalling pathway’ were highly enriched for genes whose expression was upregulated between 6 h and 24 h pi (Fig. S3b).

### Validation of the RNA-seq data by quantitative PCR

**Figure 5.**
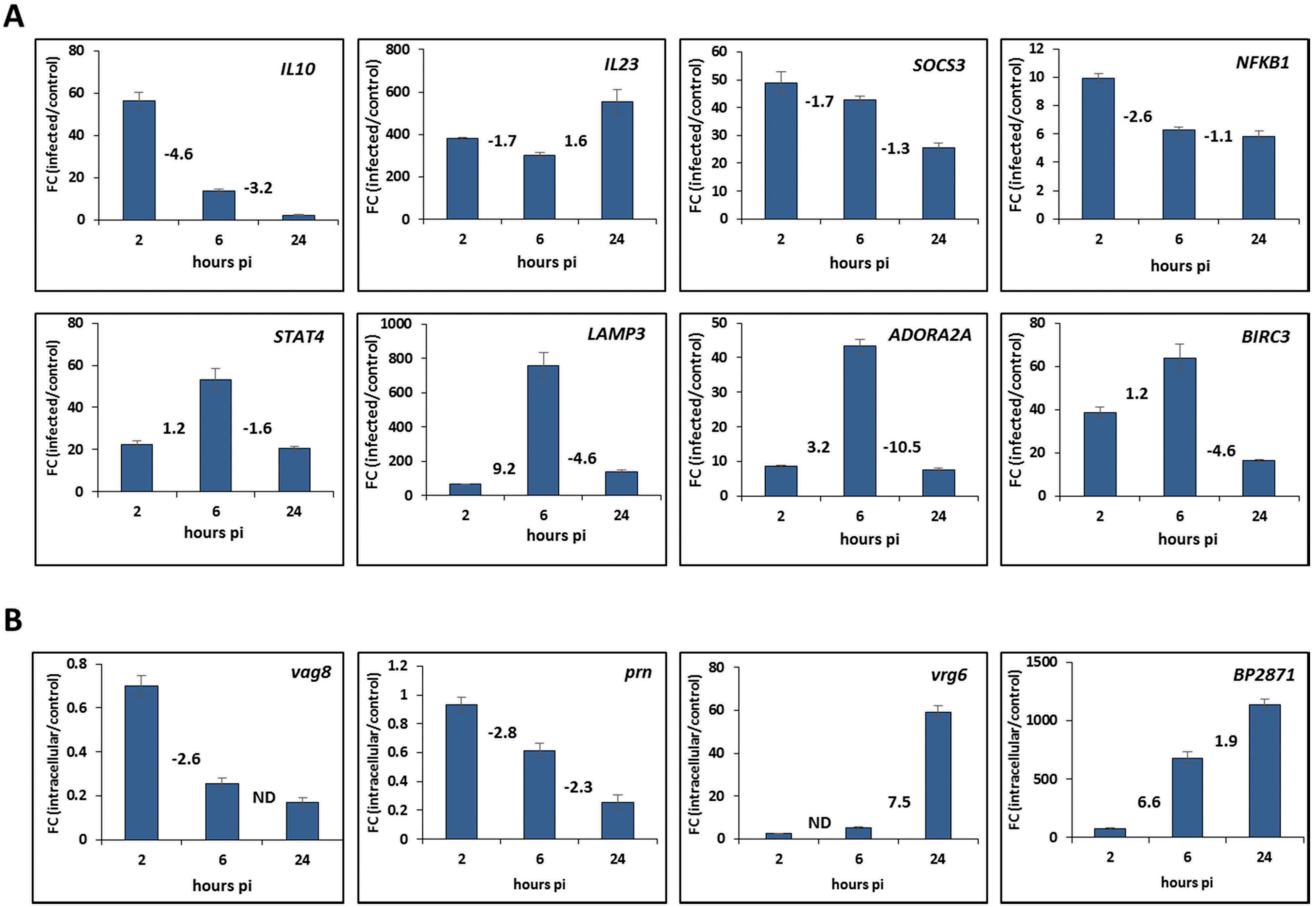
Validation of the RNA-seq results with quantitative PCR. (A) RT-qPCR analysis was performed to assay the relative expression profiles of *IL10, IL23, SOCS3, NFKB1, STAT4, LAMP3, ADORA2A* and *BIRC3* genes in infected THP-1 macrophages. Relative gene expression was compared between infected and uninfected macrophages harvested 2, 6 and 24 h post-infection (pi). (B) RT-qPCR analysis was performed to assay the relative expression profiles of *vag8, prn, vrg6* and *BP2871* genes in intracellular *B. pertussis* cells. Relative gene expression was compared between intracellular and unexposed bacteria 2, 6 and 24 h post-infection (pi). Fold change (FC) values are means (bars) ± standard deviations (error bars) from three biological replicate experiments. Values depicted between the bars denote the fold changes in expression of the specific gene between corresponding time points deduced from RNA-seq results determined 2, 6 and 24 h pi. ND, not determined in the corresponding analysis.

To validate the gene expression profiles obtained from RNA-seq analysis, the expression of selected THP-1 genes was assayed by RT-qPCR method. Comparative RT-qPCR analysis of infected and uninfected cells performed at all three time points allowed us also to confirm temporal changes in gene expression profiles deduced from RNA-seq results. As shown in Fig. 5A, results of quantitative PCR analysis of eight selected genes of different functional categories were in very good agreement with RNA-seq data. Moreover, temporal changes in the gene expression deduced from RNA-seq results are also concordant with the outcome of quantitative PCR analysis. We could, for example, verify that expression of *IL10* or *SOCS3* genes is reduced during the infection or that expression of *STAT4, LAMP3, ADORA2A* and *BIRC3* genes peaks 6 h pi.

### *Changes in gene expression profiles of intracellular* B. pertussis *cells*

Additionally, as an important part of this project, we tested the suitability of our infection protocol for parallel monitoring of the pathogen gene expression responses. Therefore, at this stage we did not fully focus on DE analysis of intracellular *B. pertussis* cells, nevertheless, we quantified abundant bacterial transcripts in samples of infected macrophages to monitor their expression profiles in the course of infection. Expectedly, more than 99% of the reads could be mapped to the human genome while the number of *Bordetella*-specific informative reads (those mapping to protein-coding genes) in samples harvested 6 h (≈ 10 000 reads/sample) and 24 h pi (≈ 6 500 reads/sample) decreased when compared to samples harvested 2 h pi (≈ 38 000 reads/sample; Fig. S1) and thereby mirrored reduced numbers of viable intracellular *Bordetella* cells recovered from macrophages in the course of infection (from 2.1 x 10^6^/well at 2 h pi to 1.0 x 10^6^/well at 6 h pi and 1.5 x 10^5^/well at 24 h pi). In spite of low sequencing yield (see Table S3 for numbers of mapped reads), we asked whether we could compare the gene expression profiles of intracellular bacteria between time points T2 and T1 and between time points T3 and T2. This analysis identified 281 and 55 significantly (│log_2_FC│ ≥ 1; adjusted p-value < 0.1) differentially expressed *B. pertussis* genes, respectively (Table S4). Among the genes strongly induced during infection were ferrous iron transporter *ftrA*, several genes implicated in sulphur metabolism including transcriptional factor *iscR*, cysteine desulfurase *iscS*, and cysteine dioxygenase *BP2871* and genes involved in vitamin B6 metabolism and homoeostasis (*BP1920* and *BP1921*) (Table 1). Of note, large number of genes encoding transcriptional regulators including *BP0135, BP0823, BP1319, BP2216*, sigma factors *rpoE* and *rpoH* and RNA chaperone *hfq* were overexpressed during the infection.

**Table 1.** List of selected *B. pertussis* genes upregulated in the course of infection^a.^

Gene ID	log_2_FC T2/T1	log_2_FC T3/T2	Name	Function
BP0135	ND	3.68	*BP0135*	LysR transcriptional regulator
BP0726	4.02	*0.95*	*BP0726*	ABC transporter
BP0823	ND	3.34	*BP0823*	transcriptional regulator
BP1152	2.09	4.01	*ftrA*	ferrous iron transport
BP1319	2.75	ND	*BP1319*	GntR transcriptional regulator
BP1320	2.67	1.76	*BP1320*	pyridoxal homoeostasis protein
BP1321	2.05	1.66	*pdxK*	pyridoxine kinase
BP1736	ND	2.55	*BP1736*	OsmY-like protein
BP1798	*1.18*	2.96	*iscR*	Fe-S transcriptional regulator
BP1799	1.69	2.73	*iscS*	cysteine desulfurase
BP2193	1.13	1.35	*hfq*	RNA chaperon
BP2216	3.12	1.80	*BP2216*	MarR transcriptional regulator
BP2437	*1.16*	1.56	*sigE*	sigma factor RpoE
BP2468	ND	2.90	*vrg6*	virulence repressed gene
BP2871	2.72	*0.90*	*BP2871*	cysteine dioxygenase
BP2782	ND	2.41	*BP2782*	lipoprotein
BP3213	1.49	1.46	*gloB*	glyoxalase II
BP3748	2.33	1.71	*BP3748*	sigma factor RpoH

Values which did not pass the statistical significance but showed similar trend are shown in italics. ND, not determined in the respective analysis.

On the other side, expression of several virulence genes such as *cyaDE, tcfA, prn, vag8, brkA* and genes within *fim, ptx*/*ptl* and T3SS-specific *bsc*/*btr* operons was significantly reduced in the course of infection (Table 2).

**Table 2.** List of selected *B. pertussis* genes downregulated in the course of infection^a.^

Gene ID	log_2_FC T2/T1	log_2_FC T3/T2	Name	Function
BP0015	−1.77	−1.68	*rpoB*	sigma factor RpoB
BP0016	−1.45	−2.10	*rpoC*	sigma factor RpoC
BP0762	−1.62	ND	*cyaD*	transport protein CyaD
BP0763	−1.65	ND	*cyaE*	transport protein CyaE
BP1054	−1.48	−1.18	*prn*	pertactin
BP1119	−2.46	ND	*fim2*	fimbriae Fim2
BP1201	*−0.75*	−3.27	*tcfA*	tracheal colonization factor TcfA
BP1251	−1.75	ND	*BP1251*	aerolysin
BP1876	ND	−1.72	*bvgR*	regulator of virulence BvgR
BP2241	−5.99	ND	*bscC*	type III secretion protein BscC
BP2244	−5.41	ND	*bscF*	type III secretion protein
BP2248	−5.35	ND	*bscI*	type III secretion protein
BP2256	−5.79	ND	*bscU*	putative secreted protein Bsp22
BP2262	−1.81	ND	*bopD*	type III secretion protein
BP2315	−1.39	ND	*vag8*	autotransporter Vag8
BP3282	−7.08	ND	*atpB*	ATP synthase subunit A
BP3494	−1.17	−1.44	*brkA*	autotransporter BrkA
BP3785	ND	−3.28	*ptxD*	pertussis toxin subunit 4 precursor
BP3786	ND	−3.28	*ptxE*	pertussis toxin subunit 5 precursor
BP3787	ND	−3.53	*ptxC*	pertussis toxin subunit 3 precursor
BP3790	ND	−2.71	*ptlC*	pertussis toxin transport protein PtlC
BP3791	ND	−1.85	*ptlD*	pertussis toxin transport protein PtlD

Values which did not pass the statistical significance but showed similar trend are shown in italics. ND, not determined in the respective analysis.

In support, the expression of *bvgR* regulatory gene which is required for expression of virulence genes was also reduced upon infection while the expression of virulence repressed gene *vrg6* was increased in the course of infection. Importantly, the outcome of quantitative PCR analysis of *vag8, prn, vrg6* and *BP2871* genes was fully in agreement with DE analysis of *B. pertussis*-specific reads determined in samples of infected macrophages (Fig. 5B). Indeed, as inferred from RNA-seq data (Tables 1 and 2), the expression of *vag8* and *prn* virulence genes was decreased in the course of infection while the expression of *vrg6* and *BP2871* genes was increased and these trends were clearly confirmed by RT-qPCR analysis. Notably, when compared to unexposed control, the expression of cysteine dioxygenase gene *BP2871* was increased more than 70-fold already 2 hours pi.

**Figure 6.**
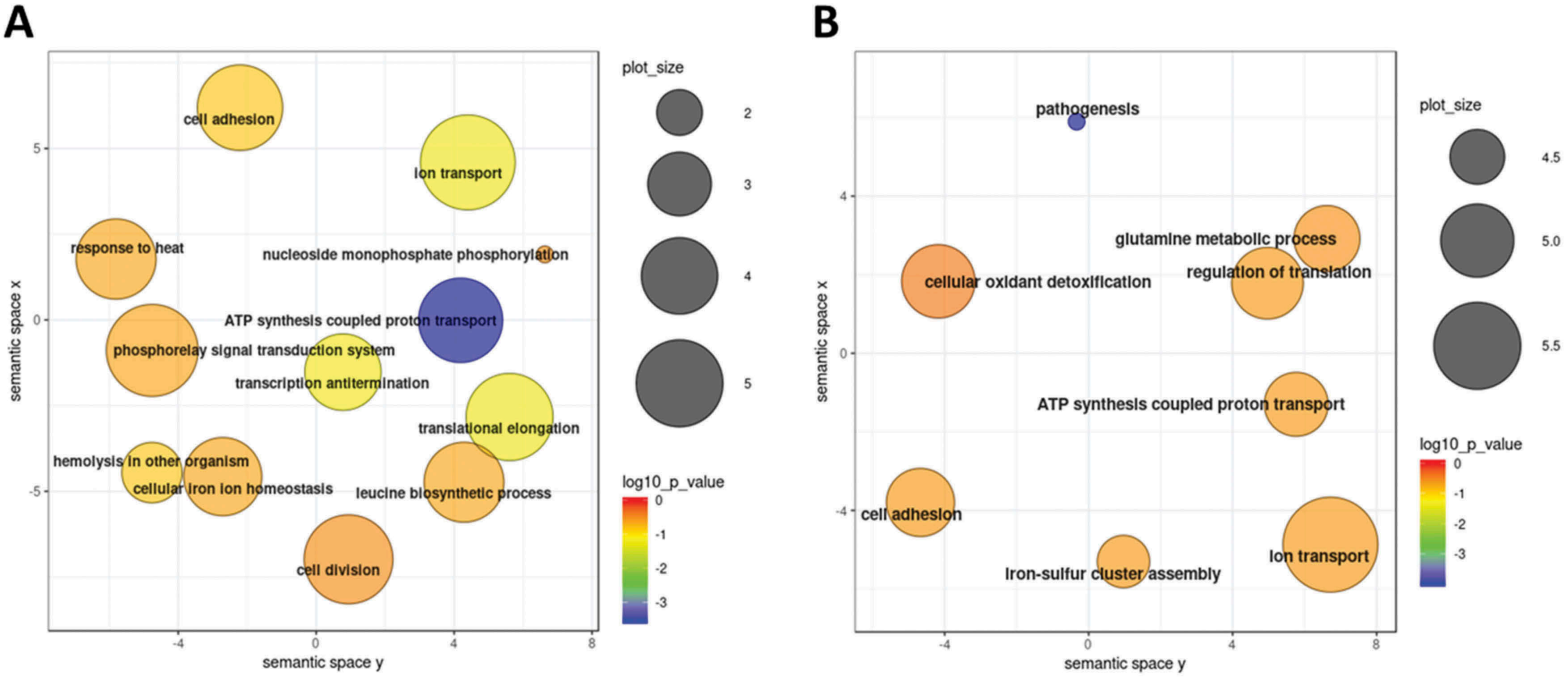
GO term enrichment analysis of *B. pertussis* genes modulated during infection. Significantly enriched terms from the domain ‘Biological processes’ were identified within gene sets either upregulated (A) or downregulated (B) between the time points T2 and T1. Results were summarized and visualized by REVIGO as a scatter plot. Circle size encodes number of genes associated with respective category, colours encode the significance level of the enrichment.

GO term enrichment analysis of genes significantly modulated between time points T2 and T1 revealed several affected cellular processes. As shown in Fig. 6A, within the set of genes which were significantly upregulated, processes such as ‘Cell redox homoeostasis’, ‘Transmembrane transport’, ‘Phosphorylation’ and ‘Oxidation-reduction process’ were highly enriched. On the other side, genes belonging to ‘Ribosome biogenesis’, ‘Dephosphorylation’, ‘Oxidation-reduction process’, ‘Pathogenesis’ and ‘ATP synthesis coupled proton transport’ terms were enriched among the transcripts which were significantly downregulated (Fig. 6B). Although only very limited number of genes displayed differential expression levels between time points T3 and T2, biological processes such as ‘Phosphorelay signal transduction’ and ‘Regulation of transcription’ were enriched for upregulated genes while ‘Pathogenesis’, ‘Proteolysis’ and‘Cell adhesion’ terms were among the processes enriched for downregulated genes (data not shown).

## Discussion

This study represents the first RNA-seq-based attempt to assess the global transcriptomic response of human phagocytes triggered by infection with *B. pertussis* cells. So far the transcriptional profiling of *B. pertussis* interactions with host cells was studied predominantly in mouse model and was focused either on the host [33,34] or the pathogen [35,36]. Nevertheless, mouse is not a natural host of *B. pertussis* and in our study we were interested in specific interaction with human macrophages rather than with complex mouse immune system. To avoid donor-to-donor variation observed in experiments with primary macrophages, we used the THP-1 monocyte-derived cells, a popular cell model for immune modulations studies [37] which has also been shown to be a good surrogate for primary cells in experiments with *B. pertussis* [21]. Profiling of human respiratory epithelial cells [38] and THP-1 macrophages [21] following *B. pertussis* infection was studied using DNA microarrays and quantitative PCR, respectively, however, global RNA-seq analysis of transcriptional responses of human phagocytic cells to *B. pertussis* cells has not been performed as yet. We have applied the dual RNA-seq approach allowing for analysis of transcriptomic profiles in both host and pathogen [39]; however, the major focus was set on the host side. To minimize the possible bias coming from additional treatment of samples delaying the RNA isolation (enrichment of the sample for bacterial RNA, time-consuming separation of infected and uninfected macrophages by flow cytometry), the macrophages were instantly lysed and used for isolation of the total RNA. This approach, however, brought some possible limitation to our study. First, as expected from previously published data [19] and our preliminary results, the numbers of viable intracellular bacteria dropped during infection and consequently, reduced relative amounts of isolated bacterial RNA combined with only a moderate sequencing depth limited the statistical power to call significantly differentially expressed genes for all but highly expressed bacterial genes. Second, we were analysing rather heterogeneous population of phagocytes composed of infected cells and cells which were either not infected or were infected but already cleared the pathogen. We accepted these limitations in order to develop an easy to control and reproducible model of infection. Indeed, obtained results clearly indicate that macrophages underwent substantial reprogramming of their transcriptome upon infection. Macrophages, like other immune cells, react to bacterial infection with common gene expression programmes [29,40]. One of the important roles of immune cells encountering the pathogen is to attract and activate other immune cells by secretion of cytokines and chemokines. Interestingly, we observed induction of both pro- and anti-inflammatory cytokines which is in line with previous reports demonstrating that TLR4-dependent recognition of *B. pertussis*-associated products triggers mixed immune response in the host [41,42]. For example, the highly expressed *IL-23, IL-1A, IL-6, CCR7, CCL2, CCL3, CXCL8, CD80* and *CD86* genes belong to set of genes characteristic for induction of M1 macrophage polarization and promoting pro-inflammatory Th1 and Th17 immune responses [43,44]. On the other side, increased expression of *IL-13* and *IL-10* cytokine genes inducing M2 programme in macrophages suggests that Th2 response is also mounted in infected macrophages. Notably, such a mixed immune response was also detected in mouse lungs after primary *B. pertussis* infection [34] and also in primary macrophages infected with several other pathogens [45–47]. Nevertheless, while the expression of *IL-23* remained highly increased in infected cells during the infection, expression of *IL-10* culminated at 2 h pi and then it gradually dropped. Importantly, these expression profiles of *IL23, IL10* and several other genes were clearly confirmed by quantitative PCR analysis. Of note, our results are in line with already documented observation that *B. pertussis*-infected monocyte-derived dendritic cells promote synthesis of IL-23 but not of IL-12 [48]. IL-23 is an IL-12-like heterodimer sharing the p40 subunit with IL-12 and was shown to play different roles in shaping of the immune response [49]. Besides its role in mounting of Th1 response, it is one of the essential factors required for the expansion of a memory T cell population, which is characterized by the production of IL-17 (Th17) [44]. Importantly, both Th1 and Th17 cells contribute to natural immunity induced by infection with *B. pertussis* in mice [50]. Also baboons, previously infected with *B. pertussis*, possess strong specific Th1 and Th17 memory [51]. Interestingly, IL-23/IL-17 axis plays a substantial role also in the development of chronic inflammations causing a variety of allergic and autoimmune diseases such as rheumatoid arthrosis, inflammatory bowel disease and asthma [52]. Based on environmental observations, Rubin and Glazer proposed a hypothesis that subclinical *B. pertussis* colonization is an important cause of asthma and diseases of allergic sensitization [53]. Therefore, it is tempting to speculate whether the paroxysmal cough, a hallmark of pertussis disease, could also result from strong pro-inflammatory IL-23 production and expansion of Th17 cells.

To prevent phagocytic killing, extracellular *B. pertussis* cells produce a very potent adenylate cyclase toxin which can disrupt cell signalling in macrophages and eventually induce apoptosis [24,25,54]. Nevertheless, our data suggest that upon internalization of *B. pertussis* cells, the expression of several genes involved in apoptotic programme is modulated which may result in prevention of cell death and permitting the pathogen to persist within the immune cells. Interestingly, antiapoptotic signalling was observed also in human respiratory epithelial cells infected with *B. pertussis* and seems to be a molecular signature of *B. pertussis* infections [38]. Indeed, majority of serpins, a group of serine protease inhibitors which are known to control an array of biological processes including inflammation [55], was significantly upregulated upon infection. Among them, SERPINB3 and SERPINB4 were reported to inhibit apoptosis in THP-1 macrophages infected with *T. gondii* [56]. Likewise, the expression of *BIRC3* (*cIAP2*) gene was highly increased in infected cells. BIRC3 is a member of the inhibitor of apoptosis family of proteins that inhibit cell death by interfering with the activation of caspases [57] and, of note, its expression was found to be increased upon *B. pertussis* infection of human bronchial cells [38].

Remarkably, within the genes strongly induced upon infection we also identified set of factors which were reported to be hijacked by several intracellular pathogens to escape from host immunity. For example, suppressors of cytokine signalling (SOCSs) proteins SOCS3 and SOCS2 belonging to important family of inflammatory response regulators [58] were shown to augment the infection with *M. tuberculosis* and *S. enterica* [59,60]. Highly induced expression of *SOCS3* in infected macrophages is also in line with previous experiments, showing increased expression of *SOCS3* gene in *B. pertussis*-infected THP-1 macrophages [21]. Similarly, increased expression of *IDO1, LAMP3* and *MARCKS* genes in infected cells may correlate with attenuation of innate immune response and increased intracellular survival as it was shown for *Mycobacterium leprae* infection of monocytes [61], *S. typhimurium* infection of HeLa cells [62] and *Burkholderia thailandensis* infection of THP-1 macrophages and epithelial A549 cells [63], respectively.

GO term and KEGG pathway enrichment analyses were applied to identify molecular signatures of macrophage response to *B. pertussis* infection. Our results showed that several signalling and metabolic pathways were significantly modulated during the infection. Challenge with *B. pertussis* (6 h pi) led among others to downregulation of mTOR and insulin signalling pathways, fatty acid metabolism, TCA cycle and oxidative phosphorylation while NOD-like receptor and chemokine signalling pathways were induced. Of note, downregulation of insulin signalling pathway may correlate with reported *B. pertussis*-induced hyperinsulinemia in human and mice [64]. Interestingly, apoptosis, Toll-like receptor and Jak-STAT signalling pathways were concomitantly up- and downregulated. These data suggest that besides modulation of signalling pathways controlling the immune response, *B. pertussis* has also profound effect on host metabolism [65].

One of the important outcomes of our study was the finding that a significant number of bacterial reads can be obtained from infected macrophage cells without enrichment steps. Yet, we are aware that our data must be interpreted with caution and corroborated in larger study yielding substantially increased number of mapped reads. Nevertheless, in this study, we could identify several *Bordetella* genes which were differentially expressed between 2 and 6 h pi and/or between 6 and 24 h pi and validate observed effects for selected genes by quantitative PCR. Among those induced during infection were genes involved in iron transport and iron-sulphur cluster assembly indicating that intracellular *B. pertussis* cells face iron scarcity and oxidative stress. These results are in line with previous reports showing that iron acquisition and transport systems and iron-sulphur cluster sensors contribute to fitness of *B. pertussis* cells during *in vivo* infections [35,36,66,67]. Within the genes displaying reduced expression during infection were also those involved in pathogenesis. The expression of *bvgR* regulator of virulence was reduced during the infection and, consequently, the expression of several virulence factors was also downregulated. This is in contrast with *in vivo* data obtained previously in mouse model of respiratory tract infection where expression of majority of virulence factors was increased during the infection of lungs [35,36,68]. Our observations indicate that opposed to *B. pertussis* cells isolated from mouse organs and facing systemic immune response, the intracellular *B. pertussis* cells switch from Bvg^+^ to Bvg^−^ phase during the intramacrophage phase. We assume that the Bvg^+^ phase plays a less significant role in the intracellular survival of *B. pertussis* and that avirulent Bvg^−^ phase can be beneficial to the pathogen as it was shown for closely related *B. bronchiseptica* [69,70]. Interestingly, Bvg^−^ mutants have been shown to accumulate among persistent *B. pertussis* cells within the upper respiratory tract of experimentally infected rhesus monkeys [71]. Furthermore, multiple metabolic pathways are upregulated in the Bvg^−^ phase and potentially help to overcome the environmental and nutritional stress [72]. Recently, we have shown that Bvg-repressed small regulatory RNA RgtA is involved in control of transport of glutamate, a key metabolite in *B. pertussis* physiology [73]. In line with these observations, the expression of virulence repressed gene *vrg6* was induced during the infection. Of note, increased expression of *vrg6* gene was observed also in *B. pertussis* cells recovered from lungs of intranasally infected mice [74]. While this gene was one of the first described virulence repressed genes in *B. pertussis* [75], its cellular function remained unclear. We suggest that Vrg6 protein belongs to family of PXP(V) proteins forming potassium channels [76,77] as it contains five PXP(V) motifs and two transmembrane domains (Fig. S4). These proteins control the activity of the pores in response to different stimuli such as changes in pH or membrane potential [78]. Considering the possible effects of harsh macrophage environment on bacterial cell wall integrity, the increased expression of the *vrg6* gene seems to be biologically relevant.

Collectively, observed changes in the gene expression profiles suggest that intracellular *B. pertussis* cells actively adapt to intramacrophage environment and respond to bactericidal activities triggered by THP-1 cells. As mentioned above, large number of host genes involved in macrophage polarization, signalling, immunosuppression and apoptosis were overexpressed upon infection. It seems that interplay between macrophages and intracellular *B. pertussis* cells may result in alternative polarization of macrophages and induction of the processes that augment survival of the bacteria within phagocytic cells. Nevertheless, strongly modulated expression of dozens of cytokines and chemokines suggests that some of the observed effects result from cell-to-cell signalling within the population of infected macrophages. Importantly, this study indicates that *in vitro* expressions profiles of several genes and pathways in both human host cells and pathogen are in line with *in vivo* data obtained with mouse model of infection [34–36,66], although our data suggest higher importance of avirulent phase in intramacrophage infection than previously thought. Nevertheless, we are aware of certain limitations of our study and it is evident that we need to perform additional experiments to delineate the *B. pertussis*-specific activities modulating the host immune response and macrophage-specific responses induced by *B. pertussis* infection in order to clarify the importance of the intracellular phase for the infectious cycle of *B. pertussis*.

## Materials and methods

### Growth and differentiation of THP-1 cells

Monocytic THP-1 cells (obtained from ATCC) were grown in suspension in 75 cm^2^ flasks in Roswell Park Memorial Institute (RPMI) 1640 medium (Sigma) supplemented with 10% heat-inactivated foetal bovine serum (FBS, Sigma) at 37°C in a humidified incubator (5% CO_2_). For differentiation into macrophages, the THP-1 cells were seeded in 6-well plates (4 x 10^6^ per well) and stimulated by the addition of 10 nM phorbol 12-myristate 13-acetate (PMA, Sigma) for 72 h followed by resting for 24 h in RPMI medium without PMA.

### *Cultivation of the* B. pertussis *cells*

*Bordetella pertussis* reference strain Tohama I was grown on Bordet-Gengou (BG) agar plates (Difco) supplemented with 15% sheep blood for 3 to 4 days at 37°C. For liquid cultures, bacteria were grown in Stainer-Scholte (SS) medium supplemented with 0.1% cyclodextrin (Sigma) and 0.5% casamino acids (Difco) at 37°C. For infection assays, the *B. pertussis* cells were grown overnight in SS medium to mid-exponential phase of growth (OD_600_ ≈ 1.0) and diluted in RPMI medium to appropriate cell density.

### Infection of THP-1-derived macrophages

Differentiated THP-1 macrophages were infected with *B. pertussis* cells diluted in RPMI medium at multiplicity of infection of 100 bacteria per macrophage (4 x 10^8^ per well). The plates were then centrifuged for 3 min at 600 g to facilitate the interaction of bacteria with macrophages. After 1 h of incubation (37°C; 5% CO_2_), nonadherent bacteria were removed by washing and remaining extracellular bacteria were killed by incubation in RPMI medium containing 100 μg/ml of polymyxin B sulphate (Sigma) for 1 h (37°C; 5% CO_2_). No viable bacteria were detected by direct plating of cell culture supernatants on BG agar. From this time point (T1), corresponding to 2 hours pi (1 h of co-incubation of *B. pertussis* cells with macrophages and 1 h of polymyxin B treatment), the concentration of the antibiotic was kept at 20 μg/ml which was sufficient to prevent the repopulation of RPMI medium with released intracellular bacteria (data not shown). At indicated times (2, 6 and 24 h pi; time points T1-T3), the infected cells were intensely washed with prewarmed phosphate-buffered saline (PBS) and directly lysed. In parallel, samples of uninfected cells were treated in the same way and served as controls. In addition, at each time point, the infected macrophages from separate well were lysed with sterile water containing 0.01% Triton X-100 and the lysates were plated onto BG agars to assess the numbers of viable intracellular bacteria. At each time point, three biological replicates of infected and uninfected macrophages as well as of unexposed bacteria cultivated in RPMI medium were harvested for transcriptomic and quantitative PCR analyses.

### RNA isolation

Macrophages were lysed with 0.5 ml of TRI reagent (Sigma) and total RNA was isolated from lysates according to the manufacturer´s protocol. Removal of DNA was achieved by treatment of samples with TURBO DNA-free kit (Thermo Fisher Scientific). RNA quality and quantity were determined by agarose gel electrophoresis and using the Nanodrop 2000 device (Thermo Fisher Scientific). Furthermore, the RNA quality was assessed at sequencing facility (Vienna Biocenter Core Facility, NGS unit) on an Agilent 2100 Bioanalyzer. All samples displayed RNA integrity numbers higher than 9.

### Library preparation, sequencing and read mapping

Ribosomal RNAs of the host and pathogen were simultaneously depleted with the Ribo-Zero Gold rRNA Removal Kit from Illumina. Libraries were prepared using NEBNext® Ultra™ II DNA Library Prep Kit for Illumina and sequenced on an Illumina HiSeq 2500 platform using HiSeqV4 chemistry with single-end 50-base-pair reads. FastQC (https://www.bioinformatics.babraham.ac.uk/projects/fastqc; version 0.11.8) was used to perform quality control checks on raw Illumina reads in FASTQ format. A single transcriptome generated by combining human transcriptome (Ensembl GRCh38) and *B. pertussis* Tohama I transcriptome was used to map and quantify the reads using Salmon quantifier (version 0.11.3) [79]. *B. pertussis* transcriptome was produced from NCBI Assembly GCF 000195715.1 using gffread (https://github.com/gpertea/gffread). After quantification with Salmon, human and bacterial read counts per gene were separated for subsequent differential expression analysis. RNA-seq data from the sequencing runs were deposited at the European Nucleotide Archive under project accession number PRJEB33395.

### Differential expression (DE) analysis

Prior to DE analysis, unwanted variation caused by batch effects or library preparation was removed from the samples using RUVs correction method from RUVseq (version 1.16.0) [80]. DE was assessed using gene read counts as inputs and analysis was performed by EdgeR package (version 3.24.1) [81]. Human genes with at least five uniquely mapped reads in at least two replicates were considered with default k = 1 using the upper-quartile normalization. Bacterial genes with at least 10 uniquely mapped reads (average of the triplicate) at least at one time point were considered with default k = 1, using a weighted trimmed mean of M-values (TMM) normalization. DE analysis for samples collected at time point T2 versus control was evaluated only for human genes, DE analysis of samples collected at time points T2 versus T1 and T3 versus T2 was determined for both human and *B. pertussis* genes.

### MSF analysis

The DE analysis results generated by edgeR were used to identify modulated sub-graphs using Modulated Sub-graph Finder (MSF). Cell signalling interactions were filtered from Reactome functional interactions version 2016 (https://reactome.org/download-data) for only direct interactions, which was used as a second input for MSF tool [82]. The parameters used for MSF were extension 2 and merging 2. The network file containing the identified interactions produced by MSF was imported and visualized by Cytoscape (version 3.6.1). KEGG pathway enrichment was generated using StringApp (version 1.4.2).

### GSEA analysis

The Gene Set Enrichment Analysis was done for GO and KEGG term enrichment using R package ‘GSEABase’ (version 1.44.0). For each comparison, the complete list of significant DE genes (adjusted p-value < 0.05) was separated into two groups, one composed of genes with positive log fold change (log_2_FC > 0) and the second composed of genes with negative fold change (log_2_FC < 0). Top 20 enriched ‘Upregulated’ and ‘Downregulated’ gene sets were selected for the visualization.

### *GO term enrichment for* B. pertussis

The gene ontology (GO) terms per gene were deduced using blast2go [83]. Each GO term which was associated with more than one gene in the gene set was tested for enrichment compared to the whole transcriptome by applying the Fisher Exact test. Afterwards, determined p-values were corrected for multiple testing by the method of Benjamini and Hochberg [84]. The GO term lists were condensed, summarized and visualized using the Revigo tool [85].

### Quantitative PCR

To validate gene expression profiles of infected THP-1 macrophages and intracellular *B. pertussis* cells, total RNA isolated for RNA-seq analysis from triplicate samples of infected macrophages 2, 6 and 24 h pi was used. As controls, total RNA was isolated from triplicates of uninfected macrophages and unexposed bacteria cultivated in parallel in RPMI medium. RT-qPCR analysis of both host and pathogen transcript levels was performed as described earlier [86]. Briefly, duplicates of 1 µg of total isolated RNA treated with TURBO DNA-free kit (Thermo Fisher Scientific) were reverse transcribed into cDNA following the manufacturer’s instructions in a 25-μl reaction using the MMLV Reverse Transcriptase (Promega). Quantitative PCR (RT-qPCR) was performed on Bio-Rad CFX384 Touch™ instrument using HOT FIREPol® Eva®Green qPCR Mix Plus™ (Solis Biodyne), 200 nmoles of each primer and 40 ng of reverse transcribed RNA in a 12-μl qPCR reaction with an initial step at 95°C for 12 min, followed by 40 cycles of 95°C for 15 s, 60°C for 25 s, 72°C for 30 s.  For THP-1 macrophages, the *ACTB* gene was used as the reference gene to assess relative gene expression levels in infected and uninfected cells. For *B. pertussis*, the *rbfA* gene was used as the reference gene to assay the relative gene expression levels in intracellular and unexposed bacteria. Primers used to quantify THP-1 and *B. pertussis* genes (Table S5) were analysed for secondary structures and for cross-dimers in primer pairs. Relative expression levels were quantified using amplification efficiency values [87]. To validate RNA-seq data, relative expression levels of selected genes were compared between infected and uninfected THP-1 cells and between intracellular and unexposed bacteria at each time point.
