# Supplementary material for: Transcriptional profiling of human macrophages during infection with Bordetella pertussis
Source: RNA Biol. 2020 Feb 19;17(5):731–42. doi: 10.1080/15476286.2020.1727694 (PMC7237194; doi:10.1080/15476286.2020.1727694)
Supplement: Supplemental Material [file krnb-17-05-1727694-s001.zip › Supplementary information/Petrackova et al Suppl figures.pptx]

## Slide 1
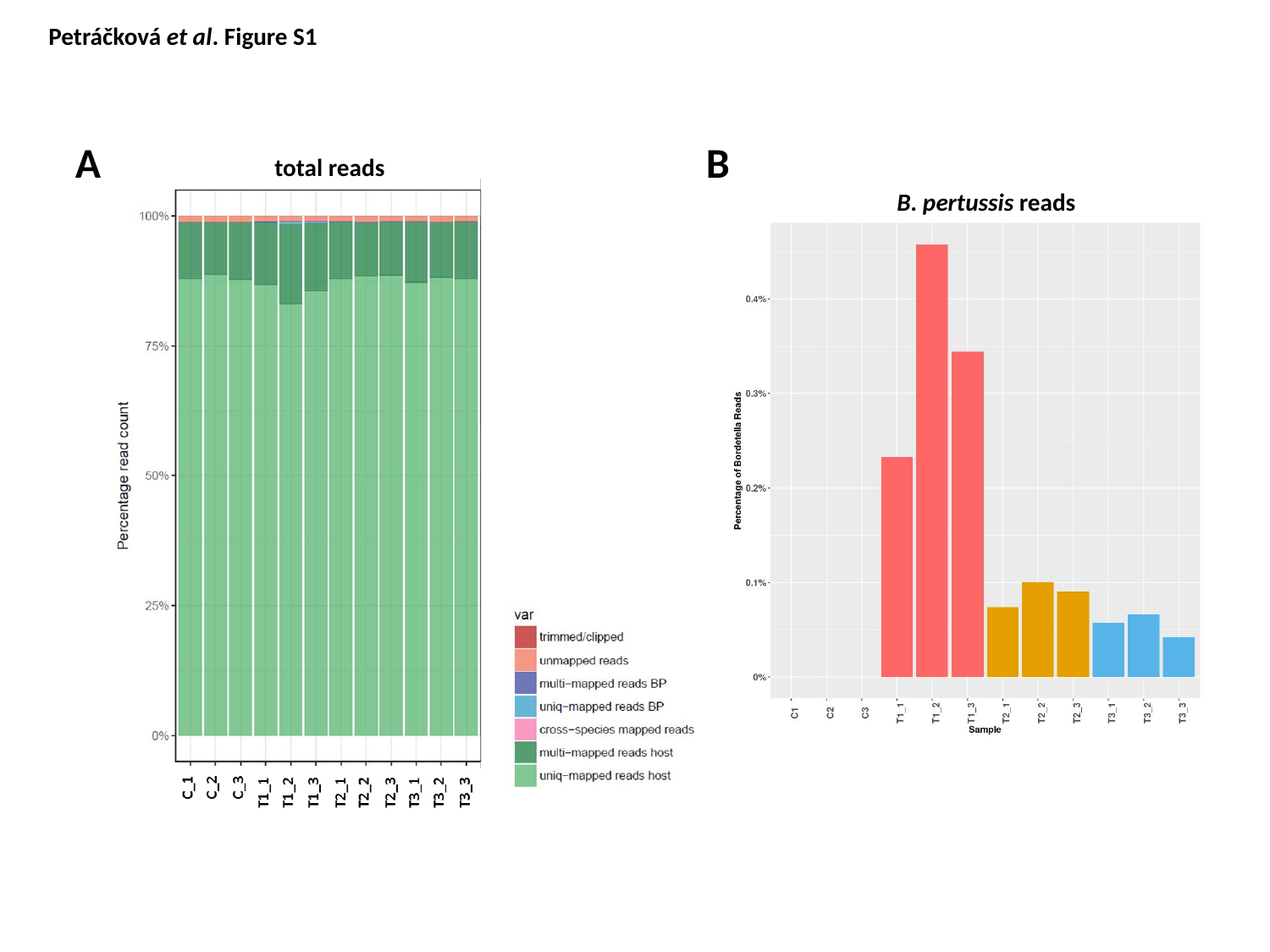

Petráčková et al. Figure S1
B
A
total reads
B. pertussis reads

## Slide 2
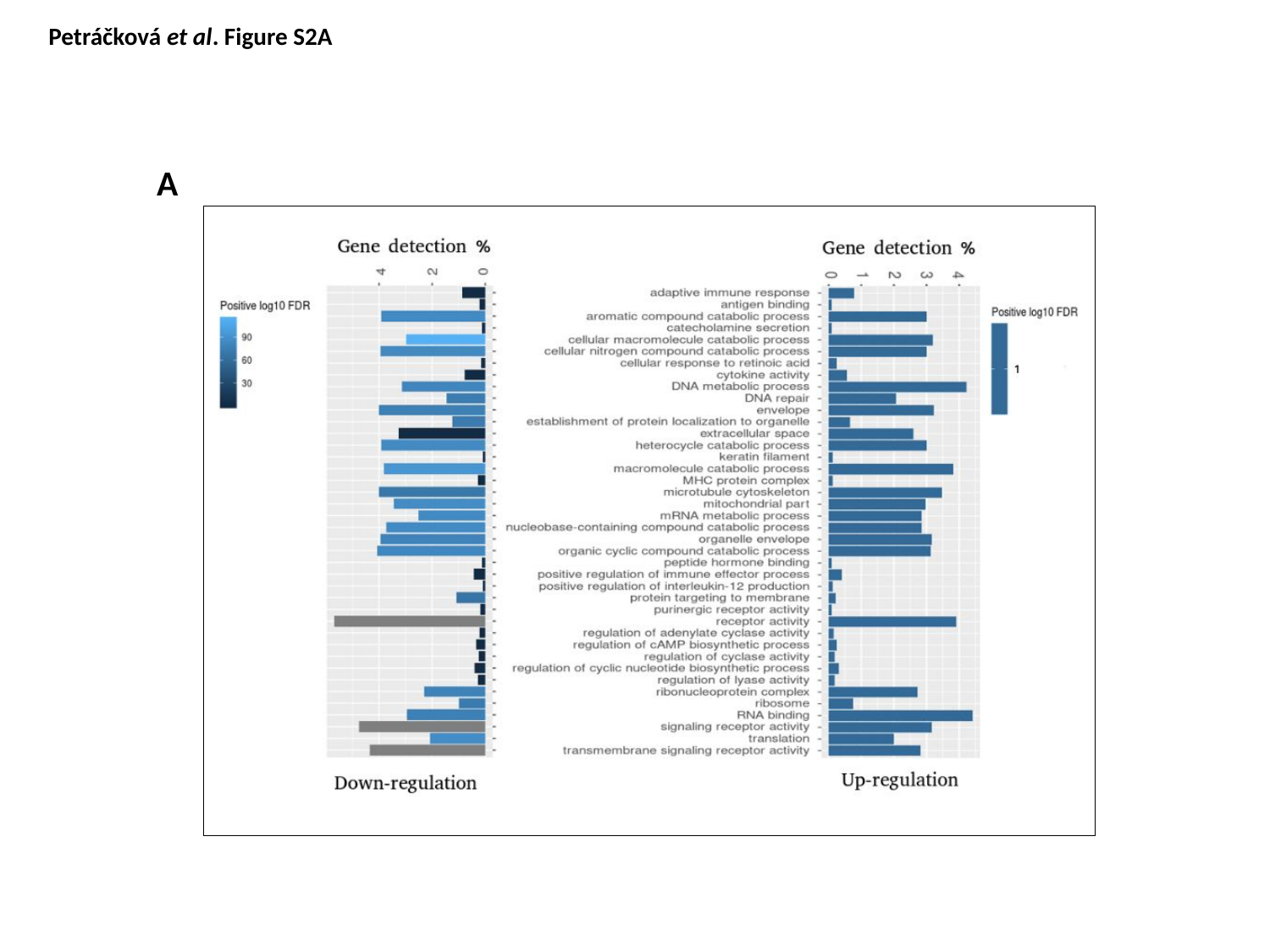

Petráčková et al. Figure S2A
A

## Slide 3
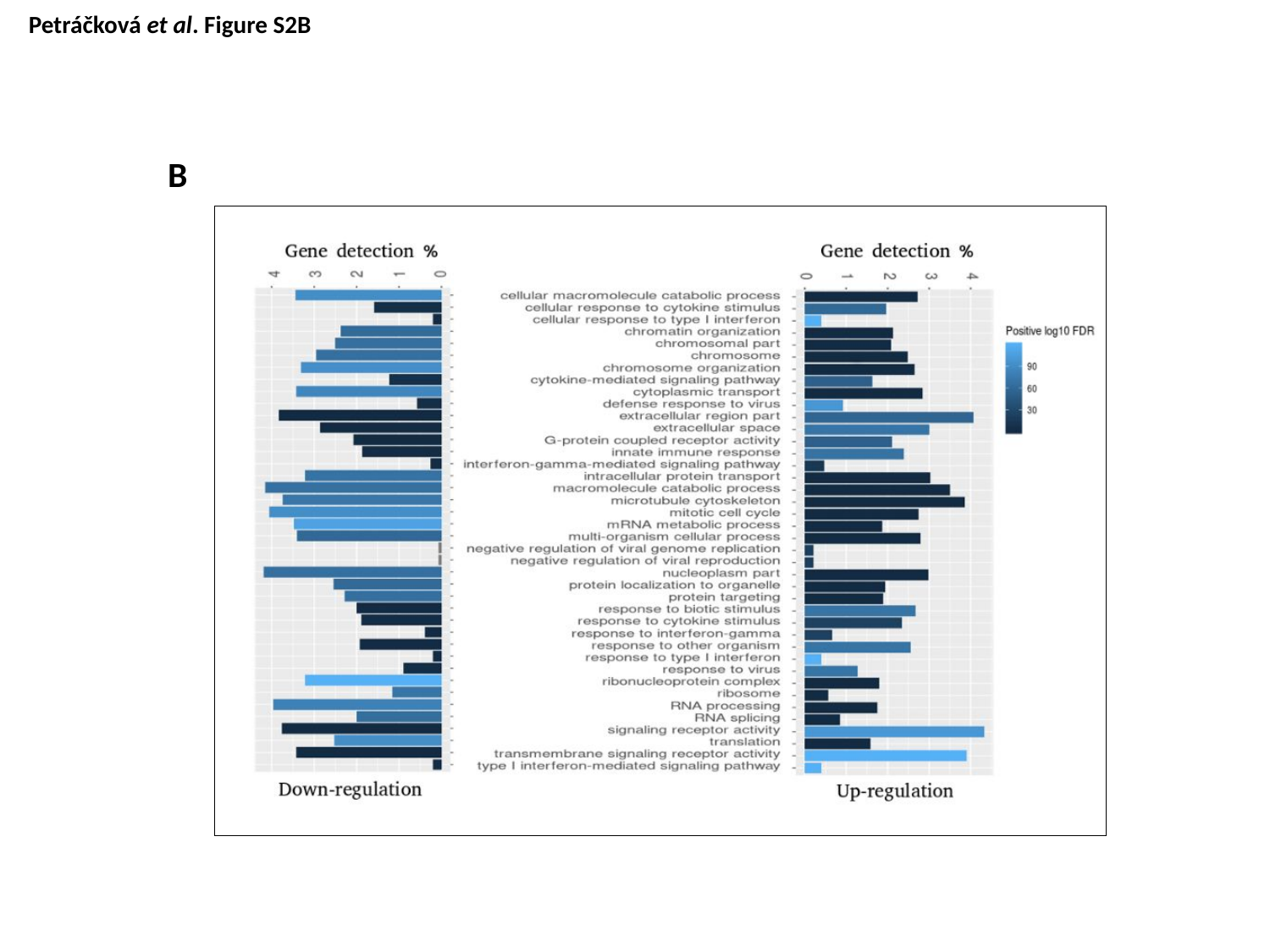

Petráčková et al. Figure S2B
B

## Slide 4
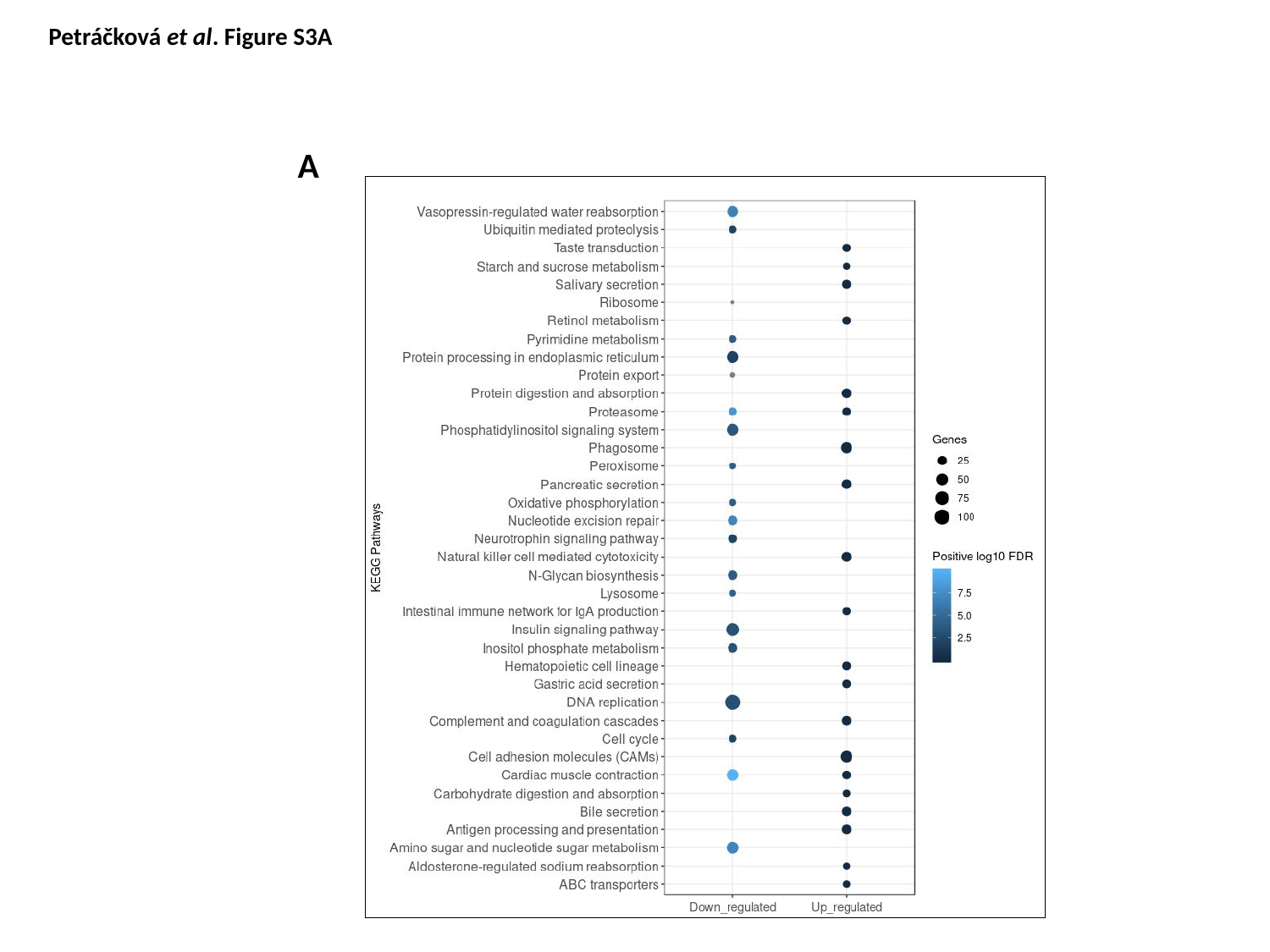

Petráčková et al. Figure S3A
A

## Slide 5
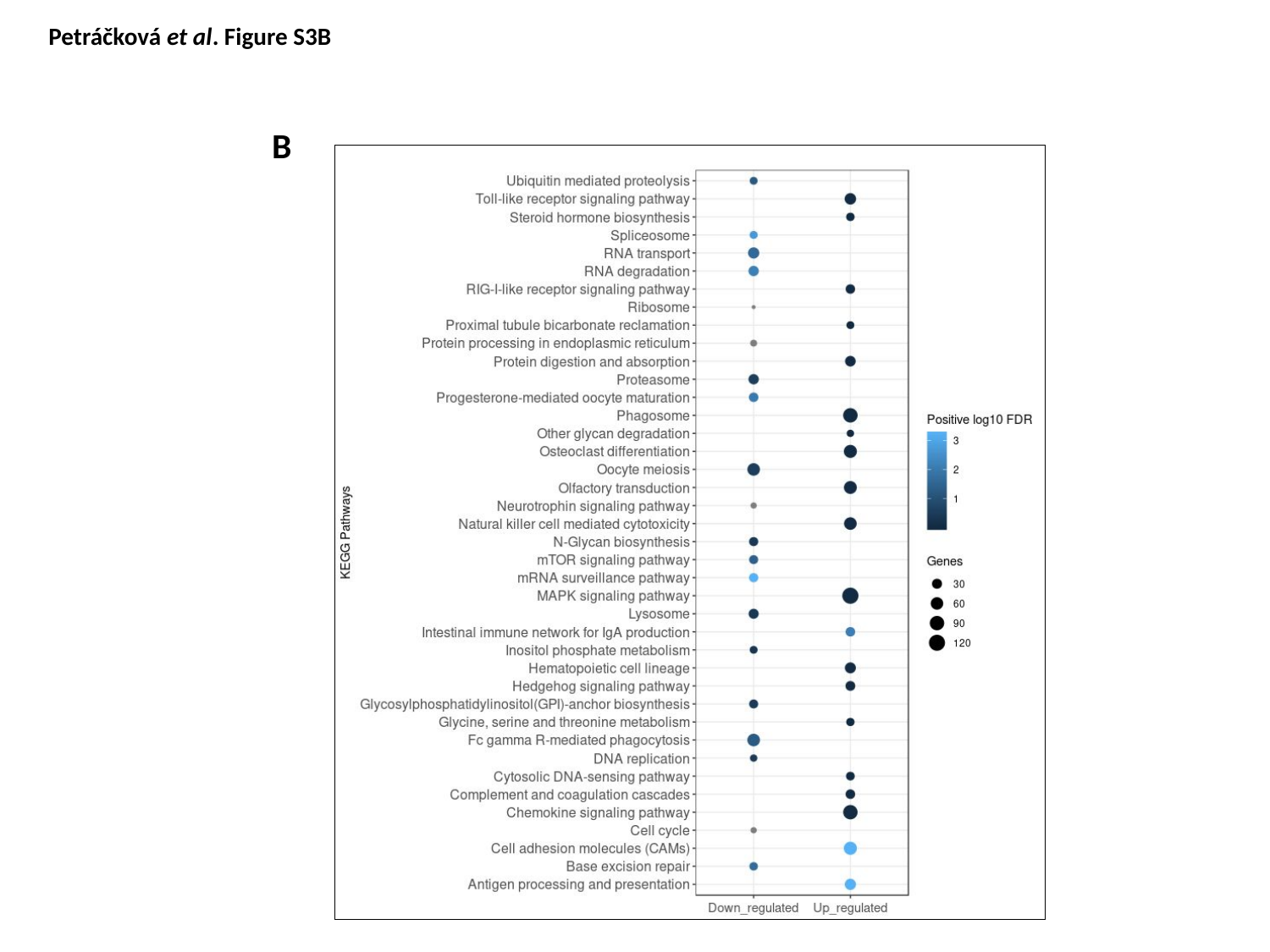

Petráčková et al. Figure S3B
B

## Slide 6
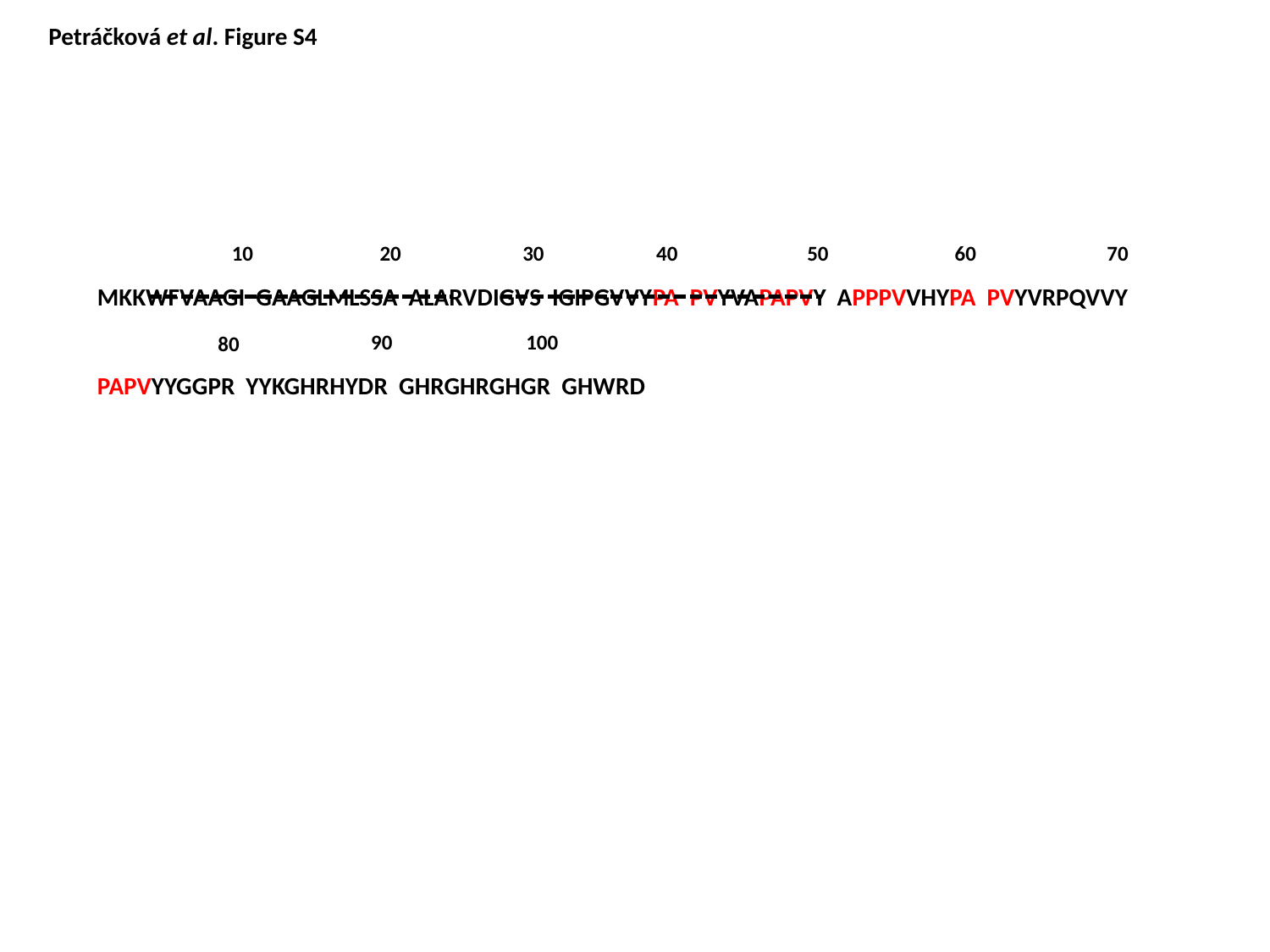

Petráčková et al. Figure S4
MKKWFVAAGI GAAGLMLSSA ALARVDIGVS IGIPGVVYPA PVYVAPAPVY APPPVVHYPA PVYVRPQVVY PAPVYYGGPR YYKGHRHYDR GHRGHRGHGR GHWRD
10
20
30
40
50
60
70
100
90
80
