# Supplementary material for: Transcriptional profiling of human macrophages during infection with Bordetella pertussis
Source: RNA Biol. 2020 Feb 19;17(5):731–42. doi: 10.1080/15476286.2020.1727694 (PMC7237194; doi:10.1080/15476286.2020.1727694)
Supplement: Supplemental Material [file krnb-17-05-1727694-s001.zip › Supplementary information/Supplementary figure legends.docx]

**Supplementary figure legends**

**Supplementary Figure S1.** Analysis of the mapped reads. (A) Plot showing distribution of all reads determined in uninfected (C) and infected macrophages (T1-T3). (B) Plot showing percentage of *B. pertussis*-specific informative reads determined in infected macrophages.

**Supplementary Figure S2.** GSEA of GO terms enriched for genes differentially expressed in the course of infection. GSEA analysis was applied to identify enriched GO terms within sets of genes either downregulated (log_2_FC ≤ 0) or upregulated (log_2_FC ≥ 0) between the time points T2 and T1 (A) or between the time points T3 and T2 (B). The bars depict the percentage of genes in the specific GO term and shades of blue indicate positive log_10_ of FDR range. The grey bars indicate that this GO term was not significantly enriched within the corresponding gene set. Top 20 enriched gene sets were selected for visualization.

**Supplementary Figure S3.** KEGG pathway enrichment for THP-1 genes downregulated or upregulated during the infection. Graph depicts pathways modulated either between the time points T2 and T1 (A) or between the time points T3 and T2 (B). The top 20 enriched pathways with a p-value < 0.05 are shown in the figure.

**Supplementary Figure S4.** Amino acid sequence of the Vrg6 protein. Putative PXP(V) motifs are shown in red, two predicted transmembrane helices (aa 4-23 and 28-50) are underlined.
